# Pharmacological Modulation of Smooth Muscles and Platelet Aggregation by *Psidium cattleyanum*

**DOI:** 10.1155/2020/4291795

**Published:** 2020-10-08

**Authors:** Hafiz Muhammad Abdur Rahman, Khaled Ahmed Saghir, Muhammad Sajjad Haider, Usman Javaid, Muhammad Fawad Rasool, Faleh Alqahtani, Imran Imran

**Affiliations:** ^1^Department of Pharmacology, Faculty of Pharmacy, Bahauddin Zakariya University, Multan 60800, Pakistan; ^2^Department of Pharmacy, Southern Punjab Institute of Health Sciences, Multan, Pakistan; ^3^Department of Pharmacy Practice, Faculty of Pharmacy, Bahauddin Zakariya University, Multan 60800, Pakistan; ^4^Department of Pharmacology and Toxicology, College of Pharmacy, King Saud University, Riyadh 11451, Saudi Arabia

## Abstract

Traditionally, in the Southern Asian countries, *Psidium cattleyanum* is a widely used plant for the management of various ailments such as gastrointestinal, respiratory, and cardiac disorders, but it lacks proof on a scientific basis, and therefore, this is the major emphasis of the current research work. Crude extract of *Psidium cattleyanum* (Pc.Cr) was preliminary analyzed for the presence of different classes of bioactive molecules. The aqueous and dichloromethane fractions of Pc.Cr were subjected to *in vitro* and *in vivo* studies. It was applied at variable concentrations (0.1–10 mg/ml) to isolated rabbit jejunum to investigate spasmolytic effect. Concentration dependent curves of calcium were constructed to check the calcium channel antagonistic activity. For the evaluation of tracheorelaxant activity, isolated tracheal tissue was treated with High-K^+^ (80 mM) and carbachol (CCh) and then challenged cumulatively with Pc.Cr. To study the antidiarrheal effect of the plant extract, castor oil-induced diarrhea model was adopted. For evaluation of the hypotensive effect of Pc.Cr, it was given intravenously to preanesthetized normotensive rats, and the response was recorded using pressure transducer. Platelet rich plasma was used for the assessment of the antiplatelet activity when challenged with purinergic and adrenergic agonists. Concentration-dependent inhibition of spontaneous and High-K^+^ mediated contractions in isolated jejunum was observed by the application of Pc.Cr. Contractions induced in isolated tracheal tissue by High-K^+^ and CCh were inhibited by application of Pc.Cr to these tissues. Similarly, application of Pc.Cr to High-K^+^ and phenylephrine (PE) treated aortic strips resulted in vasodilation. Platelet aggregation inhibition was shown by Pc.Cr against adenosine diphosphate (ADP) only. The antidiarrheal effect was observed as a reduction in the total number of feces in Pc.Cr-treated mice when given castor oil. Dose-dependent hypotension was seen in normotensive rats when treated with Pc.Cr intravenously. This study showed the spasmolytic, tracheorelaxant, vasodilator, platelet aggregation inhibitory, antidiarrheal, and hypotensive activities of *P. cattleyanum* which may be due to the blockage of calcium channels, but the involvement of any other pathway cannot be ignored.

## 1. Introduction

*Psidium cattleyanum* (*P. cattleyanum*) is an ornamental aromatic tree and known as Jeju guava, Cattley guava, strawberry guava, and Chinese guava. It belongs to family Myrtaceae, the guava family. There are about 3,800 species and 133 genera in this family, and about 92 species fall into genus *Psidium.* Many fruits of this family are medicinally important and used in traditional medicine. Among these 92 species, *Psidium cattleyanum* is commercially important due to its utility as a fruit and food [1, 2]. Its ripe fruit is eaten as fruit and also used as flavoring agent in ice cream, beverages, and desserts [3]. In Brazil it is used as analgesic, antidiarrheal, and hepatoprotective [4]. It has been reported that its extract reduces metastasis of lung cancer [4]. It possesses anticancer [5–7], antioxidant, antimicrobial, anti-inflammatory, and antidiabetic activity [2, 8]. Anticancer activity is due to presence of beta caryophyllene [9]. Tea is prepared from its leaves or the leaves are chewed to relieve toothache, abdominal pain, and throat pain. Its leaf extract has a reputation in treating respiratory problems like cough [10], inflammation, diarrhea, infections, diabetes, cancer, and other disorders. Anti-inflammatory, antioxidant [1], hypoglycemic, and antidyslipidemic activities have been reported to be present in *P. cattleyanum* [2, 11, 12]. Its leaf extract possesses enamel demineralization activity [13]. *P. cattleyanum* has better antioxidant, antibacterial, and anti-inflammatory activities than *P. guajava.* It is a good source of vitamin C and total phenolic contents [1]. It possesses antioxidant, antimicrobial, and anticancer activities and is used in folk medicines for the treatment of diarrhea, ulcer, mouth aches, and diabetes [14, 15]. More than two hundred phytochemicals have been isolated from this plant using GC-MS technique. Most important among them are ethanol, R-pinene, (Z)-3-hexenol, (E)-beta-caryophyllene, and hexadecanoic acid, which are responsible for its aromatic fragrance [1, 9]. It has also been reported that it contains large quantity of phenolic compounds. Among them epicatechin is predominant, while vitamin C, gallic acid, anthocyanin, and carotenes are available in minor concentrations [9, 10]. Aqueous-ethanolic extract of leaves of *P. cattleyanum* had been reported to contain glycitin, glycitein, sissotrin, and ononin [16]. Different monosaccharides like glucose, galactose, mannose, xylose, arabinose, and uronic acid are also present [17]. Oil of *P. cattleyanum* contains sesquiterpenes and monoterpenes. Main sesquiterpene was beta-caryophyllene followed by alpha-humulene and alpha-thujene 1, myrcene, alpha-pinene, 1,8-cineole, epi-alpha-muurolol, alpha-cadinol, and epi-alpha-cadinol [9, 13].

Because of the wide traditional uses of *P. cattleyanum* and other members of the Myrtaceae family and the limited availability of scientific literature, this study was conducted to give pharmacological basis to some of its therapeutic uses.

## 2. Materials and Methods

### 2.1. Collection and Extraction of the Plant Material

Aerial parts of *P. cattleyanum* were randomly collected from “Bio Park” of Bahauddin Zakariya University, Multan, Pakistan, in October and shed-dried after taxonomical identification. Identification voucher number was R. R. Stewart 504. This plant material was converted into a coarse powder. One-kilogram powder was macerated in 70% methanol (aqueous methanol) with occasional shaking. After a period of 8 days, the macerated material was filtered. The filtrate was dried by using a rotary evaporator (Rotavapor, BUCHI Labortechnik AG, Model 9230, Switzerland). The obtained crude extract of *P. cattleyanum* (Pc.Cr) was stored at −40°C.

For the purpose of fractionation, aqueous solution of plant extract was prepared, and then dichloromethane was added to it in a volume similar to water. This solution was continuously shaken in a separating funnel for few minutes and left there overnight. This solution split into two separate layers, i.e., aqueous and dichloromethane layers. These layers were separated and get dried, aqueous layer as Pc.Aq (aqueous fraction) and dichloromethane as Pc.DCM (dichloromethane fraction).

### 2.2. Chemicals and Drugs

Drugs used in his study are verapamil hydrochloride, acetylcholine (Ach), carbachol (CCh), and sodium citrate. Chemicals used are KCl, CaCl_2_, MgSO_4_, KH_2_PO_4_, NaHCO_3_, MgCl_2_, glucose, NaCl, and NaH_2_PO_4_. All the chemicals used were of high purity and analytical standard.

### 2.3. Phytochemical Screening

#### 2.3.1. Total Phenolic Content

Total phenolic content of Pc.Cr was determined by the standard Folin–Ciocalteu spectrophotometric method. Briefly, 0.5 ml of extract was added to 0.1 ml of Folin–Ciocalteu reagent (0.5 N), and the contents of the flask were mixed thoroughly. Later, 2.5 ml of sodium carbonate (Na_2_CO_3_) was added, mixed, and incubated for 0.5 h. The optical density was measured at 760 nm utilizing UV-visible spectrophotometer. The total phenolic contents were expressed as mg gallic acid equivalents (GAE)/g of the extract [18, 19].

#### 2.3.2. Total Flavonoid Content

For total flavonoid content, standard aluminium chloride spectrophotometric method was used. In brief, 1 ml of *P. cattleyanum* extract at a concentration of 1 mg/ml was taken. Then, 1 ml of AlCl_3_ (10%) was added sequentially. The test solution was vigorously shaken. Absorbance was recorded at 415 nm after 30 minutes of incubation. A standard calibration plot was generated at 415 nm using known concentrations of quercetin. From calibration plot, flavonoid concentration was calculated and expressed as mg quercetin equivalent (QU)/g of the extract [20].

#### 2.3.3. GC-MS Analysis

The chemical constituents of *P. cattleyanum* extract (Pc.Cr) were determined using gas chromatography and a mass spectrometer (TurboMass, PerkinElmer, Inc., Waltham, MA, USA). The temperature was set to 40°C, followed by a 2 min hold. Then the temperature was increased at the rate of 5°C/min to a maximum of 200°C and then put on hold for 2 min. After 2 minutes, temperature was again increased up to 300°C at the rate of 5°C/min and held for another 2 min again. For the determination of chemical composition of plant extract, its mass spectra were compared with the mass spectra from the National Institute of Standard and Technology and Wiley Spectral Libraries. The mass spectra of compounds were also compared with those of similar compounds in the Adams Library and the Wiley GC/MS Library [21].

### 2.4. Animals

Balb/C mice having weight range of 20–40 g, Sprague Dawley rats (150–300 g), and locally bred rabbits, both males and females, were used for the experiments. These animals were kept under standardized conditions of diet, humidity, temperature and 12-hour light and dark cycle with free access to water. Protocols for the use of animals in this study were framed under the guidelines of Institute of Laboratory Animal Resources, Commission on Life Sciences, National Research Council (1996). Departmental ethical committee approved these protocols via no. 09/PEC/2015.

### 2.5. Ex Vivo Studies

#### 2.5.1. Study on Isolated Tissues

For ex vivo studies on isolated tissues, locally bred rabbits (*n* = 10 rabbits) were used. Rabbits were kept on starvation for 24 hours before the start of experiment but with access to water. On the day of experiment, the rabbits were sacrificed, and different tissues like jejunum, trachea, aorta, and atrium were isolated and kept in physiological salt solution [22–24].

*(1) Study on Rabbit Jejunum*. Antispasmodic effect of *P. cattleyanum* was studied by using rabbit jejunum. 2-3 cm long pieces of isolated jejunum were made after cleaning the adhering tissues like mesenteries. Each segment was left in Tyrode's solution-filled organ bath. The solution was kept on continuous perfusion with carbogen and maintained at pH 7.4 and 37°C. A weight of 1g was applied as preload, and tissue response was recorded by MLT0015 isotonic transducer which was connected with PowerLab. To equilibrate the tissue, Tyrode's solution was repeatedly changed after every 30 minutes Acetylcholine (0.3 *µ*M) was repeatedly applied to the tissue to stabilize it every 3 minutes. After stabilization of jejunum, antispasmodic response of respective plant extract was investigated by application in a cumulative fashion. Spasmolytic effect of the extract was calculated using

(1) $$\begin{array}{c} \%\text{ age relaxation}=\frac{\left(A-B\right)}{A}\times 100, \end{array}$$

where A is the control tissue response before the administration of any drug and B is the tissue response after drug administration.

Calcium plays a vital role in the contraction of smooth muscles, and this calcium comes from the extracellular fluid via calcium channels or comes from intracellular stores. Any drug which blocks these calcium channels is considered as calcium channel blocker and causes the relaxation of muscles. To investigate the involvement channel blockade in the spasmolytic potential of Pc.Cr, we construct calcium curves as described earlier by [24–27].

*(2) Tracheorelaxant Study on Isolated Rabbit Trachea*. Isolated trachea of rabbits was cut into small pieces having a size of about 3-4 mm in width, with 2-3 cartilages. These tracheal pieces were opened by cutting the cartilage in the opposite direction of smooth muscle layer. Tissue organ bath was cleaned and filled with Krebs solution, and prepared tracheal tissue was put in it. Solution in organ bath was kept on continuous perfusion with carbogen. 1 gram preload tension was applied to the suspended tissue. This tissue was kept free to equilibrate for 30–45 minutes, but the solution was replaced repeatedly. The tracheorelaxant response of test drug was observed on precontracted isolated tracheal strip with High-K^+^ and CCh via PowerLab connected isometric transducers [28, 29].

(*3) Vasorelaxant Study on Rabbit Aorta*. Aorta was isolated from rabbits and cleaned off the mesenteries. Cleaned aorta was divided into 2-3 cm small rings/strips. This aortic ring was suspended in Krebs solution-filled organ bath. It was ensured that supply of carbogen should not be interrupted throughout the experiment and temperature should be kept at 37°C. Suspended tissue was kept free for 30 minutes to equilibrate, and 2.0 gram preload tension was applied to it. In order to stabilize the tissue, it was repeatedly treated with either phenylephrine (1 *µ*M) or High-K^+^. After stabilization, tissue was cumulatively treated with plant extract to observe its vasodilator effect. Results were recorded by MLT 0201 transducer linked with PowerLab [30, 31].

*(4) Study on Rabbit Atrium*. To study the cardiac inhibitory effect of the plant extract, right atrium of the rabbits was isolated and used. A 10 mL organ bath was cleaned and filled with Krebs solution, and isolated atrium was put in it. The solution in the bath was kept on continuous perfusion with carbogen, and temperature maintained at 37°C throughout the experiment. A preload tension of 1.0 gram was applied to the atrium. Atrial contractions were recorded by PowerLab linked isometric transducer. The left atrium was allowed to equilibrate for 30 minutes with repeated changing of Krebs solution. During this time no drug was applied to the left atrium. After equilibration, the tissue was treated with acetylcholine (1 *µ*M) or isoprenaline (1 *µ*M) to check its responsiveness. Plant extract was applied to this tissue cumulatively to observe the response of the tissue, and results were calculated as percentage of the baseline response [26, 32].

#### 2.5.2. Platelet Aggregation Inhibition Study on Human Plasma

Inhibition of aggregation of platelets by the application of Pc.Cr was studied by using a previously described method [27, 33, 34]. Blood samples were collected from healthy volunteers (*n* = 5). Collected blood was centrifuged at 1,500 rpm for a period of 15 minutes, and supernatant was collected as platelet rich plasma (PRP). Remnant was recentrifuged at 4,000 rpm speed for 20 minutes, and supernatant separated and collected as platelet poor plasma (PPP). Chrono-log 490-2D aggregometer was utilized for this study. This aggregometer works on light transmission aggregometry principle. Machine was turned on and kept free to maintain the temperature. 0.23 mL of PRP and 10 *µ*L of plant extract were taken in micro cuvette and placed in PRP chamber. A spacer was attached at the base of the cuvette. Then, 10 *µ*L of ADP (5 *µ*M) or epinephrine (40 *µ*M) was added to this cuvette. 450 µL of PPP was added to another micro cuvette and kept as a reference in PPP chamber [35]. Ethical approval was granted by departmental ethical committee for this work via no. EC/03-PHD/2019, and a consent form was also signed by the volunteers.

### 2.6. In Vivo Studies

#### 2.6.1. Antidiarrheal Study

Castor oil induced diarrhea model was used to study the antidiarrheal effect of Pc.Cr as reported previously [23, 24]. Thirty-six (36) mice were equally divided into six groups and placed separately in individual cages. The 1st and 2nd groups were treated with 10 mL/kg of 0.9% NaCl and 10 mg/kg of loperamide, respectively, while the 3rd, 4th, 5th, and 6th groups were given orally Pc.Cr 50 mg/kg, Pc.Cr 100 mg/kg, Pc.Cr 300 mg/kg, and Pc.Cr 500 mg/kg, respectively. All these mice were orally treated with castor oil 10 mL/kg 60 minutes after the respective treatments and kept under observation for 6 hours. The total number of feces of each mouse were counted and analyzed.

#### 2.6.2. Hypotensive Activity

SD rats (*n* = 5) were anesthetized with intraperitoneal administration of ketamine and diazepam at a dose range of 50–80 mg/kg and 5 mg/kg, respectively. After the development of anesthesia, the animal was placed on a dissection board in an upward position. Isothermic warming cushions were utilized to maintain the temperature at 37°C. A small incision was made for cannulation of trachea, jugular vein, and carotid artery. Polyethylene tube (PE-20) was used for cannulate trachea, while jugular vein and carotid artery were cannulated with PE-50. Cannula of carotid artery was filled with heparin solution (60IU/Ml) and attached to disposable pressure transducer (MLT0699) which was attached to PowerLab for data recording. After cannulation, 0.1 mL heparin was administered to rat through jugular vein. Plant extract and standard drugs were administered in a volume of 0.1 mL which was followed by a flush of 0.1 mL normal saline. Epinephrine (1 *µ*g/kg) and acetylcholine (1 *µ*g/kg) were given to check the response of the animal to hypertensive and hypotensive drugs. After equilibration, 0.1 mL Pc.Cr was administered through jugular vein, and the response to different doses was recorded [30, 36].

#### 2.6.3. Acute Toxicity Study

15 mice were equally divided into five groups by random selection and used to observe the acute toxicity of plant extract. The 1st group of animals was treated with Pc.Cr 1.0 g/kg, the 2nd group was given Pc.Cr 3.0 g/kg, and the animals of the 3rd group were treated with 5.0 g/kg. These mice were critically observed for any physical and behavioral change as well as mortality for up to 24 hours [26].

### 2.7. Statistical Analysis

Data values are represented in the form of mean ± SEM, and EC_50_ values were determined by nonlinear regression. Two-way ANOVA followed by Tukey's test was applied to determine the significant difference among the concentrations. Hypotensive results were analyzed by Student's *t*-test, while antiplatelet and antidiarrheal results were analyzed by one-way ANOVA followed by Dunnett's test.

## 3. Results

### 3.1. Phytochemical Screening

#### 3.1.1. Total Flavonoid Contents

The total flavonoid content of *P. cattleyanum* extract (Pc.Cr) was measured with standard aluminium chloride spectrophotometric method. It was shown that Pc.Cr contained a total flavonoid content of 33.2 mg QU/g.

#### 3.1.2. Total Phenolic Contents

On the other hand, the total phenolic content of the *P. cattleyanum* extract and fractions was measured with the Folin–Ciocalteu reagent assay. It was shown that Pc.Cr contained (95.8 mg gallic acid/g).

#### 3.1.3. GC-MS Analysis

**Figure 1:**
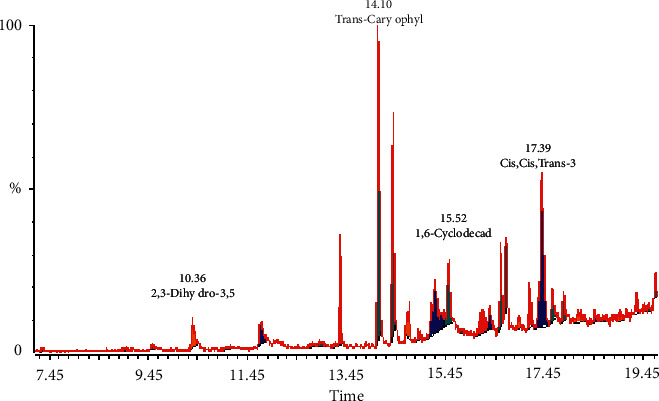
GC-MS chromatogram of Pc.Cr methanolic extract.

**Figure 2:**
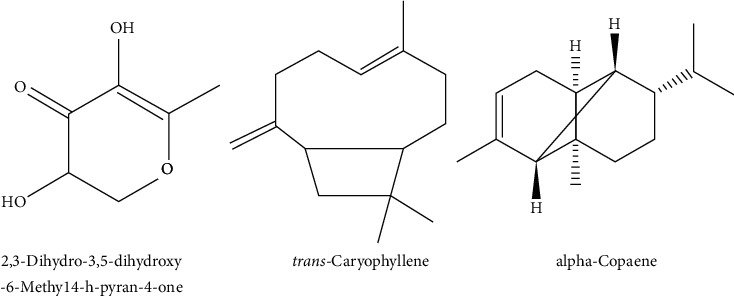
Major active constituents of *Psidium cattleyanum*.

The identified compounds of Pc.Cr, along with their retention times and area percentages, are shown in Figure 1 and Table 1. These compounds are represented in the order of their elution on the HP-INNOWax GC column (Agilent Technologies, USA). A total of 12 compounds, representing 98.99% of the total extract, have been identified. 2,3-Dihydro-3,5-dihydroxy-6-methyl 4H-pyran-4-one (17.1%) was the major abundant constituent, and caryophyllene (12.3%) was a moderate constituent. The other compounds reported in Table 1 are present in fairly good amounts. The structure of some active compounds is presented in Figure 2.

### 3.2. Ex Vivo Studies

#### 3.2.1. Effect on Rabbit Jejunum

**Figure 3:**
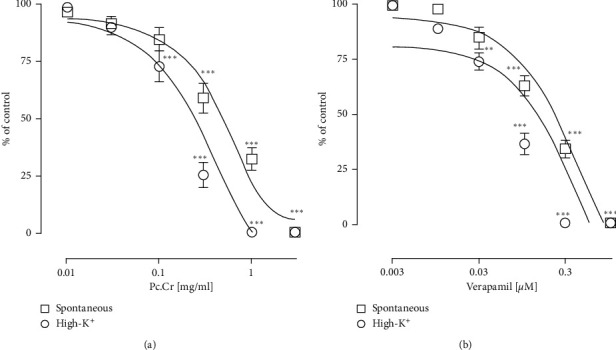
Concentration based curves of (a) Pc.Cr and (b) verapamil on isolated jejunum. Data are shown as mean ± SEM, *n* = 6–8, and analyzed by two-way ANOVA with Tukey's test. ^*∗*^*P* < 0.05, ^*∗∗*^*P* < 0.01, and ^*∗∗∗*^*P* < 0.001.

**Figure 4:**
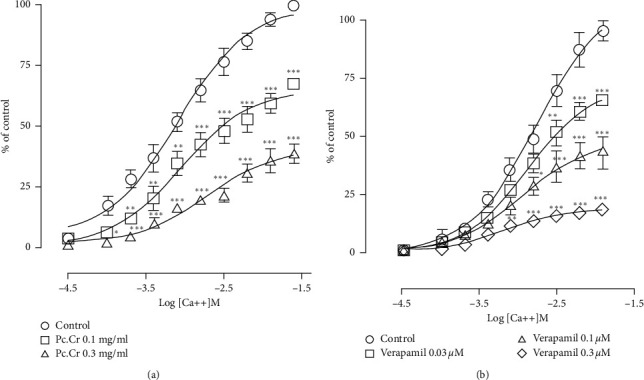
(a) Pc.Cr. (b) Verapamil showing the inhibition of concentration response curves (CRCs) of calcium on jejunum. Data values are presented as mean ± SEM of 4–6 and analyzed by two-way ANOVA with Tukey's test. ^*∗*^*P* < 0.05, ^*∗∗*^*P* < 0.01, and ^*∗∗∗*^*P* < 0.001 show a comparison of different concentrations of plant extract with respect to control.

**Figure 5:**
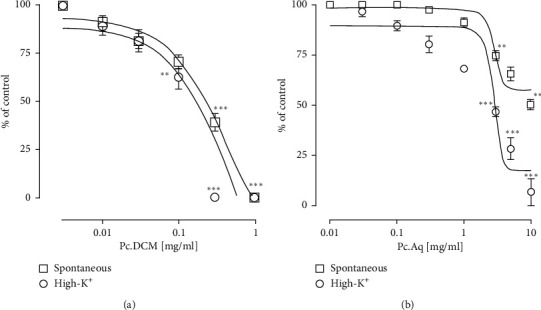
Concentration oriented effect of (a) Pc.DCM and (b) Pc.Aq on isolated jejunum. Data are shown as mean ± SEM, *n* = 5–8 and analyzed by two-way ANOVA with Tukey's test. ^*∗*^*P* < 0.05, ^*∗∗*^*P* < 0.01, and ^*∗∗∗*^*P* < 0.001.

Spontaneous and High-K^+^ induced contractile responses were inhibited by the application of Pc.Cr in a concentration-dependent way to isolated jejunums. Respective values of EC_50_ were 0.76 ± 0.11 (0.46–1.29 mg/mL) and 0.25 ± 0.14 (0.11–0.44 mg/mL). Verapamil, a standard antagonist of calcium channel, showed similar behavioral against jejunum with EC_50_ value of 0.20 ± 0.06 (0.14–0.27 *µ*mol) and 0.08 ± 0.06 (0.06–0.08 *µ*mol) (Figure 3). These results indicated the presence of calcium channel blocking potential in plant extract. For the confirmation of this activity, tissue was incubated with Pc.Cr and then treated with increasing concentration of CaCl_2_. Contractile effect of calcium with rightward shifting of CRCs was observed. Hence, the presence of calcium channel antagonist activity was proved (Figure 4). Similarly, Pc.DCM, when applied to rabbit jejunum, inhibited both normal rhythmic contractions and High-K^+^ generated contractions with EC_50_ values of 0.40 ± 0.10 (0.26–0.65 mg/mL) and 0.16 ± 0.15 (0.08–0.27 mg/mL). Application of Pc.Aq resulted in partial inhibition of spontaneously contracting jejunum, and complete inhibition of potassium induced contractions was seen. EC_50_ value was 2.86 ± 0.15 (2.21–6.41 mg/mL) (Figure 5).

#### 3.2.2. Tracheorelaxant Effect

**Figure 6:**
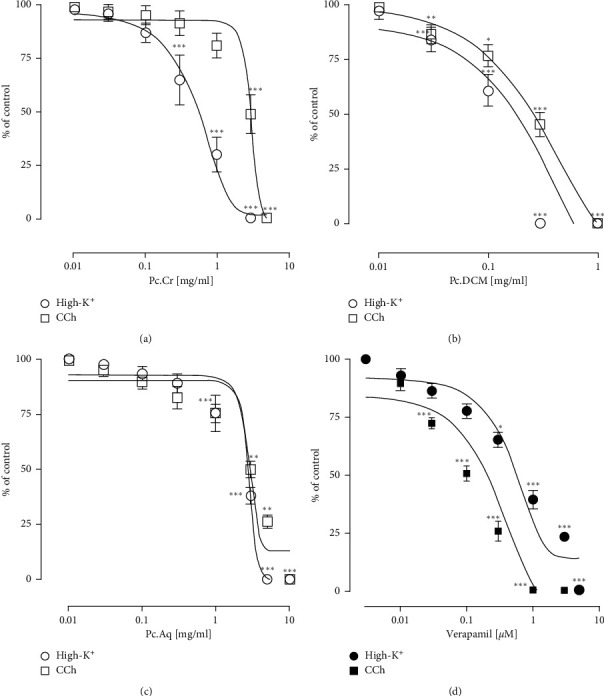
Concentration based curves of (a) Pc.Cr, (b) Pc.DCM, (c) Pc.Aq, and (d) verapamil against tracheal contractions generated by either High-K+ or CCh. Data are presented as mean ± SEM, *n* = 4–6, and analyzed by two-way ANOVA with Tukey's test. Significant *P* values, i.e., ^*∗*^*P* < 0.05, ^*∗∗*^*P* < 0.01, and ^*∗∗∗*^*P* < 0.001, represent the effect of plant extract in comparison with preceding concentration of the extract.

Tracheal contractions induced by High-K^+^ and CCh were inhibited by the application of Pc.Cr, Pc.DCM, and Pc.Aq in a concentration dependent way. These effects were similar to verapamil, but the effect of Pc.DCM was more potent than Pc.Cr and Pc.Aq. Respective EC_50_ values against High-K^+^ and CCh generated Pc.Cr contractions of 0.80 (0.62–1.07 mg/mL) and 4.99 ± 0.20 (3.29–7.83 mg/mL). For Pc.DCM, EC_50_ values were 0.15 ± 0.12 (0.08–0.21 mg/mL) and 0.56 ± 0.08 (0.40–0.80), respectively, but for Pc.Aq, EC_50_ values were 3.55 ± 0.12 (2.36–5.33 mg/mL) and 6.90 ± 0.20 (4.12–12.84 mg/mL), respectively (Figure 6).

#### 3.2.3. Effect on Isolated Rabbit Aorta

**Figure 7:**
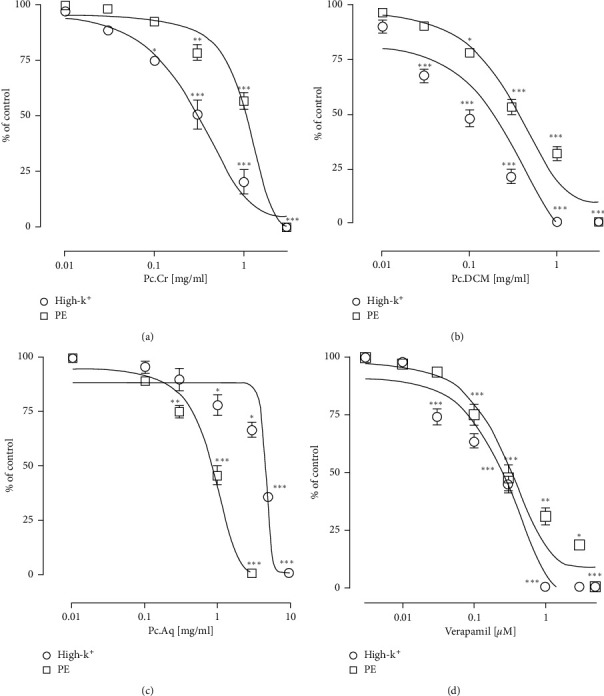
Concentration oriented vasorelaxant curves of (a) Pc.Cr, (b) Pc.DCM, (c) Pc.Aq, and (d) verapamil. Data are analyzed by two-way ANOVA with Tukey's test. ^*∗*^*P* < 0.05, ^*∗∗*^*P* < 0.01, and ^*∗∗∗*^*P* < 0.001 were considered significantly different and represent the effect of plant extract vs preceding concentration of the extract.

Pc.Cr, its fractions and verapamil when applied to High-K^+^ and PE treated isolated aortic tissues, concentration based vasorelaxant responses were seen. Respective EC_50_ values of Pc.Cr were 0.40 ± 0.09 (0.25–0.59 mg/mL) and 1.92 ± 0.10 (1.03–1.72 mg/mL). Similarly, vasorelaxant by Pc.DCM was observed at EC_50_ values of 0.11 ± 0.06 (0.05–0.13 mg/mL) and 0.52 ± 0.07 (0.36–0.76 mg/mL). Vasorelaxant effect of verapamil was observed with respective EC_50_ values of 0.20 ± 0.06 (0.14–0.29 µmol) and 0.32 ± 0.17 (0.23–0.45 *µ*mol). Pc.Aq also showed inhibitory effects at higher concentrations with EC_50_ values of 0.97 ± 0.08 (0.72–1.30 mg/mL) and 4.25 ± 1.16 (3.05–6.0 mg/mL) (Figure 7). Vasorelaxant effect of plant was more potent against High-K^+^ induced vasoconstriction than PE-induced vasoconstriction like verapamil.

#### 3.2.4. Effect on Isolated Rabbit Atrium

**Figure 8:**
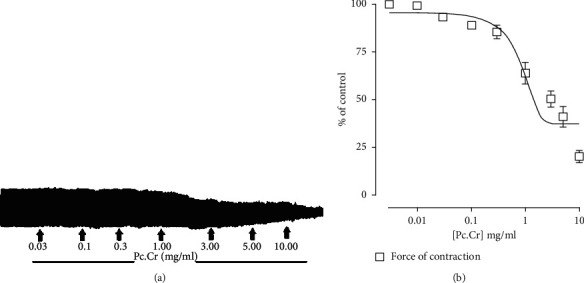
Atrial inhibition by Pc.Cr: (a) original tracings, (b) graph. Data are presented as mean ± SEM, *n* = 3–5.

Concentration dependent inhibition of force of contractions by applying Pc.Cr to isolated atrium with EC_50_ value of 2.20 ± 0.20 (1.17–4.36 mg/mL) was observed, but no effect on rate of contraction was observed (Figure 8).

#### 3.2.5. Inhibition of Platelet Aggregation

**Figure 9:**
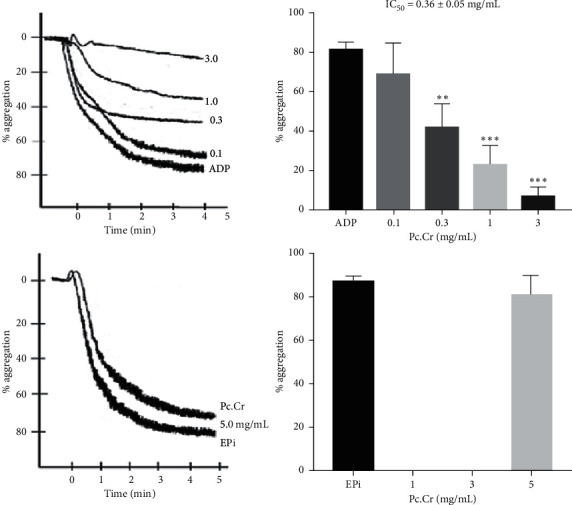
Percentage of platelet aggregation inhibition by Pc.Cr in ADP and Epi induced aggregation. Data are analyzed by one-way ANOVA and represented as mean ± SEM, *n* = 5–7. ^*∗∗*^*P* < 0.01, ^*∗∗∗*^*P* < 0.001 vs control values.

Inhibition of aggregation in platelets was shown by Pc.Cr in the presence of agonists like ADP with minimum inhibitory concentration of 0.27 ± 0.09 (0.22–0.34 mg/mL), but no inhibition was observed in the presence of epinephrine (Epi) up to dose of 5 mg/ml (Figure 9).

### 3.3. In Vivo Studies

#### 3.3.1. Antidiarrheal Activity

**Figure 10:**
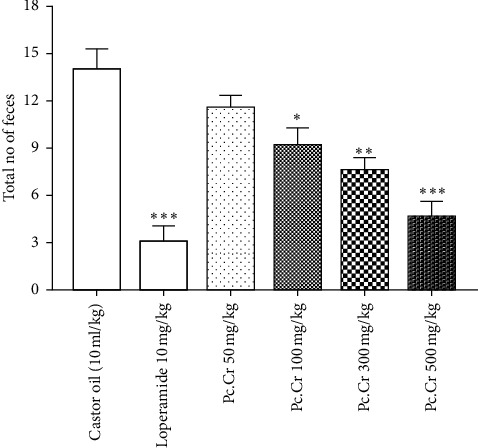
Bar graph showing decrease in fecal output of mice treated with Pc.Cr and later treated with castor oil. Data are shown as mean ± SEM, and statistical parameter of one-way ANOVA was adopted to analyze it (^*∗*^*P* < 0.05, ^*∗∗*^*P* < 0.01, ^*∗∗∗*^*P* < 0.001).

Castor oil treated mice were protected significantly by giving Pc.Cr (p.o.) in a dose range of 100 mg/kg to 500 mg/kg (*P* ≤ 0.01) as evident by decrease in total feces in a time period of six hours. The dose of 50 mg/kg showed no significant protection. Protection was observed by decrease in total numbers of feces in a time period of six hours like loperamide (10 mg/kg) (*P* ≤ 0.001). No antidiarrheal effect was observed by the administration of saline (Figure 10).

#### 3.3.2. Hypotensive Effect

Administration of Pc.Cr to normotensive rats caused reduction in blood pressure with respect to control (normotensive). Administration of Pc.Cr 1.0 mg/kg resulted in reduction of SBP to 95.67 ± 2.16 mmHg vs normotensive SBP 140.67 ± 11.61 mmHg, DBP to 71.68 ± 4.68 mmHg vs normotensive DBP 121.05 ± 21.45 mmHg, and MAP to 127.48 ± 21.18 mmHg. Similarly, Pc.Cr 3.00 mg/kg inhibited SBP to 79.86 ± 3.0 mmHg, DBP to 68.74 ± 8.71 mmHg, and MAP to 68.55 ± 6.90 mmHg compared to normotensive rats. However, Pc.Cr 10.00 mg/kg reduced SBP to 39.23 ± 2.41 mmHg, DBP to 30.71 ± 0.84 mmHg, and MAP to 34.19 ± 0.29 mmHg compared with control group (Table 2).

### 3.4. Acute Toxicity Study

No sign of toxicity was observed in any animal for a period of 24 hours.

## 4. Discussion

Smooth muscle relaxant drugs are used usually in the treatment of cardiovascular, respiratory, and gastrointestinal disorders [37]. Considering the traditional uses of *P. cattleyanum* and other members of its family in the treatment of such ailments, it was planned to explore the pharmacological insights of these traditional uses.

Spasmolytic effect of plant extract was seen when applied to isolated jejunum. These spasmolytic effects were similar to those of verapamil. Smooth muscle contraction is based on intracellular free calcium. Increase in intracellular calcium may induce contraction in smooth muscles [38, 39]. Calcium enters to the cells through voltage activated calcium ion channels or is released from sarcoplasmic reticulum [40, 41]. When potassium in high concentration (High-K^+^) applied to the tissue, contraction of smooth muscle takes place due to the opening of calcium ion channels. Increased intracellular calcium activates the contractile machinery of the smooth muscle cells. Calcium channel blockers are the drugs which block the opening of these channels and cause relaxation. Concentration dependent curves of calcium were constructed for the confirmation of calcium channel blocker potential of plant extract. It was observed that pretreatment of tissue with Pc.Cr caused the rightward shift of calcium curves with inhibition of maximum contractile response, thus confirming the presence of calcium antagonist effect of plant extract [27, 28, 42].

Pc.Cr and its fractions also showed inhibition of High-K^+^ and carbachol mediated contractions in isolated tracheal strips. Tracheorelaxant effect of Pc.DCM was more potent as compared to Pc.Aq. CCh has different mechanism of contraction from that of High-K^+^. It causes the stimulation of muscarinic receptors of M_3_ type. Muscarinic receptor stimulation causes increase in intracellular concentration of calcium through phospholipase C second messenger system, and as a result contraction takes place [34, 43]. It was observed that tracheorelaxant effect of plant extract was dominant against potassium induced contractions compared with CCh induced tracheal contraction, similar to verapamil.

PE and High-K^+^ mediated vasoconstrictions were inhibited by the application of Pc.Cr and its fractions. However, like calcium channel antagonists, inhibition against High-K^+^ was more than that against PE, again giving a clue for the presence of calcium channel blocker property in *P. cattleyanum*. As blood pressure is the product of cardiac output and peripheral vascular resistance (BP = CO × PVR), Pc.Cr was applied to isolated rabbit atrium to see its influence on cardiac output. Partial reduction in force of contraction with no change in rate of contraction was seen. Therefore, it can be said that *P. cattleyanum* selectively blocks the calcium channels of the smooth muscles but has little influence on calcium channels of cardiac muscles.

Dose dependent diarrheal protection was also seen. Reduction of blood pressure in normotensive rats treated with Pc.Cr was observed. Due to vasodilator and cardiac inhibitory properties of calcium channel antagonists, they are used in the treatment of hypertension and other ailments of cardiac system [44–46]. Flavonoids, terpenes, and saponins have been reported to possess relaxant effect on smooth muscles [38, 39]. Pc.Cr also contains these macromolecules as indicated by our phytochemical results and previous literature. Accordingly, we can say that terpenes, saponins, and flavonoids are involved in modulation of gastrointestinal, respiratory, and cardiovascular ailments. *Psidium cattleyanum* contains *trans*-caryophyllene as indicated by GC-MS analysis which is reported to possess spasmolytic and calcium channel blocker activity [47]. *trans*-Caryophyllene may be the one of those macromolecules which cause the relaxation of smooth muscles.

Platelet aggregation was blocked because Pc.Cr epinephrine (Epi) induced aggregation was not inhibited. P_2_Y_1_ and P_2_Y_12_ glycoprotein receptors exist on the surface of platelet and play a vital role in aggregation. ADP causes the stimulation of these receptors and results in the aggregation of platelets. It can be said that plant extract possesses ADP antagonistic potential which caused the inhibition of platelet aggregation [33, 35]. Calcium has a vital role in ADP mediated platelet aggregation. It has been observed that Pc.Cr possesses calcium channel antagonistic activity, which may be possibly involved in aggregation inhibition [48, 49]. It has been reported that terpenoids, tannins, and flavonoids are involved in platelet aggregation and smooth muscle relaxation [50–53]. Our phytochemical studies of Pc.Cr indicated that sesquiterpenes and flavonoids are present in it. Hence, it can be said that spasmolytic and platelet aggregation inhibition may be due to the presence of these macromolecules.

## 5. Conclusion

Spasmolytic, tracheorelaxant, vasodilator, atrial inhibitory, platelet aggregation inhibitory, diarrheal protective, and hypotensive activities of *P. cattleyanum* were observed in this study, thus giving a justification for using it in treating ailments of gastrointestinal, respiratory, and cardiovascular systems. These results of *P. cattleyanum* may be due to the blockage of voltage gated calcium channels, and presence of *trans*-caryophyllene and flavonoids may be the reason for these effects. However, the involvement of any other mechanism cannot be ignored. Platelet aggregation inhibition by Pc.Cr may be due to antagonism of P_2_Y_1_ and P_2_Y_12_ receptors. This is a preliminary research, and exploration of the exact mechanism of action and identification of active compounds from *Psidium cattleyanum* require further investigation.

## Figures and Tables

**Table 1:** GC-MS analysis of *P. cattleyanum* leaves with the identified phytoconstituents, their retention time, and percentage areas.

Compound name	Chemical formula	Molecular weight (g/mol)	RT (min)	Area %
2,3-Dihydro-3,5-dihydroxy-6-methyl 4H-pyran-4-one	C_6_H_8_O_4_	144.12	10.35	17.070
4,5-Dimethyl-4-hexen-3-one	C_8_H_14_O	126.2	11.71	9.930
Alpha-copaene	C_15_H_24_	204.36	13.33	7.480
*trans*-Caryophyllene	C_15_H_24_	204.36	14.10	12.290
Bergamotene	C_15_H_24_	204.35	14.38	9.730
Beta selinene	C_15_H_24_	204.35	14.71	4.040
Germacrene D	C_15_H_24_	204.35	15.52	4.630
(−)-Caryophyllene oxide	C_15_H_24_O	220.35	16.57	7.790
*cis,cis,trans*-3,3,6,6,9,9-Hexamethyl-tetracyclo[6.1.0.0(2,4).0(5,7)]nonane	C_15_H_24_	204.35	17.42	16.100
(1r,5s,e)-2-Methyl-4-[2,2,3-trimethyl-6-methylidenecyclohex-2-en-1-yl]but-2-enal	C_15_H_24_O	220	17.61	2.540
Caryophyllene diepoxide	C_15_H_24_O_2_	236	17.85	2.100
Tetrahydroionone	C_13_H_24_O	196.33	19.69	2.700

**Table 2:** The observed values in mm of Hg for hypotensive effect of Pc.Cr on invasive cannulated carotid artery of rats.

Treatment	SBP	DBP	MAP
Control	140.67 ± 11.61	121.05 ± 21.45	127.48 ± 21.18
Verapamil	83.45 ± 2.36	63.23 ± 2.18	69.89 ± 2.21
Pc.Cr 1.00 mg/kg	95.67 ± 2.16^*∗∗*^	71.68 ± 4.68^*∗*^	80.34 ± 3.57^*∗*^
Pc.Cr 3.00 mg/kg	79.86 ± 3.00^*∗∗*^	61.74 ± 8.71^*∗∗*^	68.55 ± 6.90^*∗∗*^
Pc.Cr 10.00 mg/kg	39.23 ± 2.41^*∗∗∗*^	30.71 ± 0.84^*∗∗∗*^	34.19 ± 0.29^*∗∗∗*^

Data values are represented in the form of mean ± SEM, *n* = 5. Student's *t*-test was applied for data evaluation. ^*∗*^*P* < 0.05, ^*∗∗*^*P* < 0.01, and ^*∗∗∗*^*P* < 0.001.

## Data Availability

The data used to support the findings of this study are available from the corresponding author upon request.

## Abbreviations

ADP: Adenosine diphosphate
CCh: Carbamylcholine or carbachol
CI: Confidence interval
CRCs: Concentration response curves
DAG: Diacylglycerol
DBP: Diastolic blood pressure
Epi: Epinephrine
EC_50_: Minimum effective concentration
High-K: High concentration of potassium (K-80 mM)
IP3: Inositol triphosphate
MAP: Mean arterial pressure
Pc.Cr: Crude extract of *Psidium cattleyanum*
Pc.DCM: Dichloromethane fraction of *Psidium cattleyanum* crude extract
Pc.Aq: Aqueous fraction of *Psidium cattleyanum* crude extract
PE: Phenylephrine
PPP: Platelet poor plasma
PRP: Platelet rich plasma
SBP: Systolic blood pressure.
